# Anti-quorum Sensing of Terminalia catappa and Murraya koenigii Against Streptococcus mutans

**DOI:** 10.7759/cureus.48765

**Published:** 2023-11-13

**Authors:** Kavitha Ramsundar, Ravindra Kumar Jain, Sankar G Pitchaipillai

**Affiliations:** 1 Orthodontics and Dentofacial Orthopaedics, Saveetha Dental College and Hospitals, Saveetha Institute of Medical and Technical Sciences (SIMATS) Saveetha University, Chennai, IND; 2 Dentistry, Saveetha Dental College and Hospitals, Saveetha Institute of Medical and Technical Sciences (SIMATS) Saveetha University, Chennai, IND; 3 Microbiology, Saveetha Dental College and Hospitals, Saveetha Institute of Medical and Technical Sciences (SIMATS) Saveetha University, Chennai, IND

**Keywords:** micro-organism, medicinal plants, white spot lesions, biofilm, quorum sensing

## Abstract

Introduction

Dental biofilm constitutes micro-organisms existing in an intercellular matrix containing organic and inorganic materials derived from saliva, gingival crevicular fluid, and bacterial products. Dental plaque biofilm inhibition by certain herbs and medicinal plants has been used as a treatment modality for the prevention of white spot lesions in orthodontic subjects. The aim of this study was to evaluate the anti-quorum sensing and anti-biofilm activity of *Terminalia catappa* and *Murraya koenigii*against *Streptococcus mutans.*

Materials and methods

Samples of dental plaque were taken from patients receiving orthodontic care. The colonies of the* S. mutans* were isolated and biochemical characterization was done. Leaf extracts of *Terminalia catappa *and *Murraya koenigii* were used in the study. Methanolic extracts were subjected to evaluation of minimum inhibitory concentration (MIC) using the broth microdilution (two-fold) method and anti-biofilm activity using the crystal violet staining method.

Results

The MIC of methanol leaf extracts of *Murraya koenigii* against *S. mutans* was noted at 0.62 mg/ml and *Terminalia catappa* at 1.25 mg/ml. At the lowest concentration of 0.03 mg and 0.01 mg methanol extract of *Murraya koenigii *had remarkably inhibited biofilm formation of 57.6% and 43.6% against *S. mutans*, respectively. *Terminalia catappa* leaf extracts did not show any anti-biofilm activity when the organisms were grown in the presence of* S. mutans*.

Conclusion

Both *Murraya koenigii *and *Terminalia catappa* had antibacterial effects against *S. mutans* and *Murray koenigii *remarkably inhibited biofilm formation by *S. mutans*.

## Introduction

Dental biofilm is defined as microorganisms existing within an intercellular matrix that consists of organic and inorganic materials derived from saliva, gingival crevicular fluid, and bacterial products [1]. Dental plaque harbors more than 500 types of different bacteria which are commonly implicated in dental caries and periodontal diseases. Dental caries is the most common disease in mankind and its prevention and treatment have been widely researched [2].

Quorum sensing (QS) is a form of intercellular communication between bacterial cells that involves the synthesis and detection of chemical signaling molecules called autoinducers [3]. QS regulates various activities, such as biofilm formation, sporulation, bioluminescence, and expression of virulence factors. White spot lesions (WSLs) are one of the most unwanted iatrogenic side effects of orthodontic treatment because they result from prolonged "undisturbed" dental plaque biofilm formation on the teeth surfaces, which is typically caused by poor oral hygiene [4]. *Streptococcus mutans* is a pathogenic microbe of dental plaque as it produces biofilms and is strongly associated with carious WSLs [5]. Competence-stimulating peptides, which are perceived by a two-component signal system, are produced by *S. mutans* as QS signals [6]. *S. mutans* uses Com-dependent QS systems to regulate genetic transformation, natural competence, colonization, sporulation, virulence, and biofilm formation [7].

*Murraya koenigii* known as kari patta or curry leaves in India belongs to the family Rutaceae and is included in Indian dishes as a condiment in the preparation of pickles, powders, and sausages and is also available as oils in markets. The branches of *Murraya Koenigii *are used to strengthen gums and teeth in traditional medicine [8]. *Terminalia catappa* has been investigated in several studies and is known to have diverse chemical composition. *Terminalia catappa* extract exhibits antioxidant, antimicrobial, anti-inflammatory, antiviral, and hepato-protective effects. It has been reported previously that ethanol extracts of *Terminalia catappa* leaves have reduced biofilm formation by *Candida albicans* [9]. Ethanolic extracts of *Murraya koenigii* leaves have demonstrated comparable antibacterial activity against *S. mutans* as chlorhexidine in a few studies [10,11]. According to reports, quorum quenching involves suppression of any one of the crucial QS signal pathways exploited for QS and preventing microbial infections [12,13].

Antimicrobial efficacy studies in the past have assessed the efficacy of plant extracts on *S. mutans* [5]. There is a need to develop innovative strategies that can inhibit* S. mutans* more effectively. One of these strategies can be using the various medicinal plants available [6]. Also, research on the anti-QS activity of medicinal plants has been rapidly increasing in recent years [14,15]. Evaluation of anti-QS and anti-biofilm activity of *Murraya koenigii* and *Terminalia catappa* against *S. mutans* can be done as both these plants have shown good antibacterial activity against* S. mutans*. This study was done to assess the anti-biofilm activity of leaf extracts of *Terminalia catappa* and *Murraya koenigii* against *S. mutans* and also establish the minimum inhibitory concentration (MIC) required to suppress plaque biofilm formation.

## Materials and methods

Study design and clinical isolates

Patients who visited for orthodontic treatment at Saveetha Dental College and Hospitals, Chennai, India in December 2022 were included in this study after obtaining their consent. Samples of dental plaque were taken from the patients receiving orthodontic care. The study protocol was approved by the Ethical Committee of Saveetha Institute of Medical and Technical Sciences (SIMATS), Chennai, India (IRB number is SRB/SDC/ORTHO-2007/22/067). With the aid of a sterile No. 23 Shepherd's hook explorer, the plaque samples were taken. The collected 10 samples were inoculated into brain heart infusion (BHI) broth (HiMedia Laboratories Pvt. Ltd., Mumbai, India) and incubated for 24 hours at 37°C. After incubation, the samples from BHI broth were streaked over Mitis Salivarius Agar (HiMedia Laboratories Pvt. Ltd., Mumbai, India) plate and then incubated for 48 hours at 37°C. The colonies of the *S. mutans* (raised, opaque, undulated, pale blue, frothy glass appearance) were isolated [16]. For further examination, the isolated colonies were kept in storage at 4°C.

Biochemical characterization of *S. mutans*

*S. mutans *were identified by the standard microbiological test [13] which included typical growth patterns on Luria-Bertani agar (HiMedia Laboratories Pvt. Ltd., Mumbai, India), colony morphology on Mitis Salivarius Agar, gram staining (HiMedia Laboratories Pvt. Ltd., Mumbai, India), catalase (HiMedia Laboratories Pvt. Ltd., Mumbai, India), oxidase (HiMedia Laboratories Pvt. Ltd., Mumbai, India), motility, urease (HiMedia Laboratories Pvt. Ltd., Mumbai, India), citrate (HiMedia Laboratories Pvt. Ltd., Mumbai, India), indole (HiMedia Laboratories Pvt. Ltd., Mumbai, India), methyl red (HiMedia Laboratories Pvt. Ltd., Mumbai, India), Voges Proskaur's (Himedia, India), nitrate reduction test (Himedia, India), gelatin hydrolysis (HiMedia Laboratories Pvt. Ltd., Mumbai, India), H2S test (HiMedia Laboratories Pvt. Ltd., Mumbai, India), lactose (HiMedia Laboratories Pvt. Ltd., Mumbai, India), sucrose (HiMedia Laboratories Pvt. Ltd., Mumbai, India), and lipid hydrolysis (HiMedia Laboratories Pvt. Ltd., Mumbai, India).

Collection of medicinal plants

*Terminalia catappa and Murraya koenigii *leaves were gathered from an orchard at the Saveetha Institute of Medical and Technical Sciences in Chennai, Tamil Nadu, India (between latitudes 13°3'26.5392"N and 80°7'21.4752"E). The leaves from *Terminalia catappa* and *Murraya koenigii* were shade-dried for a week and powdered using a motor and pestle. About 10g of powdered samples were extracted separately using 95% methanol. The extracts were concentrated using a rotary flash evaporator. The extracts were separately redissolved in DMSO (2.5%).

Evaluation of MIC

Evaluation of the MIC of the medicinal plants (*Terminalia catappa *and *Murraya koenigii*) was done using the broth microdilution (two-fold) method. The MICs of the methanolic leaf extracts employed in the experiment against *S. mutan*s were evaluated at various doses between 10 and 0.01 mg/mL. By introducing an indicator salt called 2,3,5-triphenyl tetrazolium chloride, the growth of the bacteria was seen. The MIC value was recorded as the lowest concentration with no discernible increase in bacterial growth [17,18].

Biofilm inhibition assay

**Figure 1:**
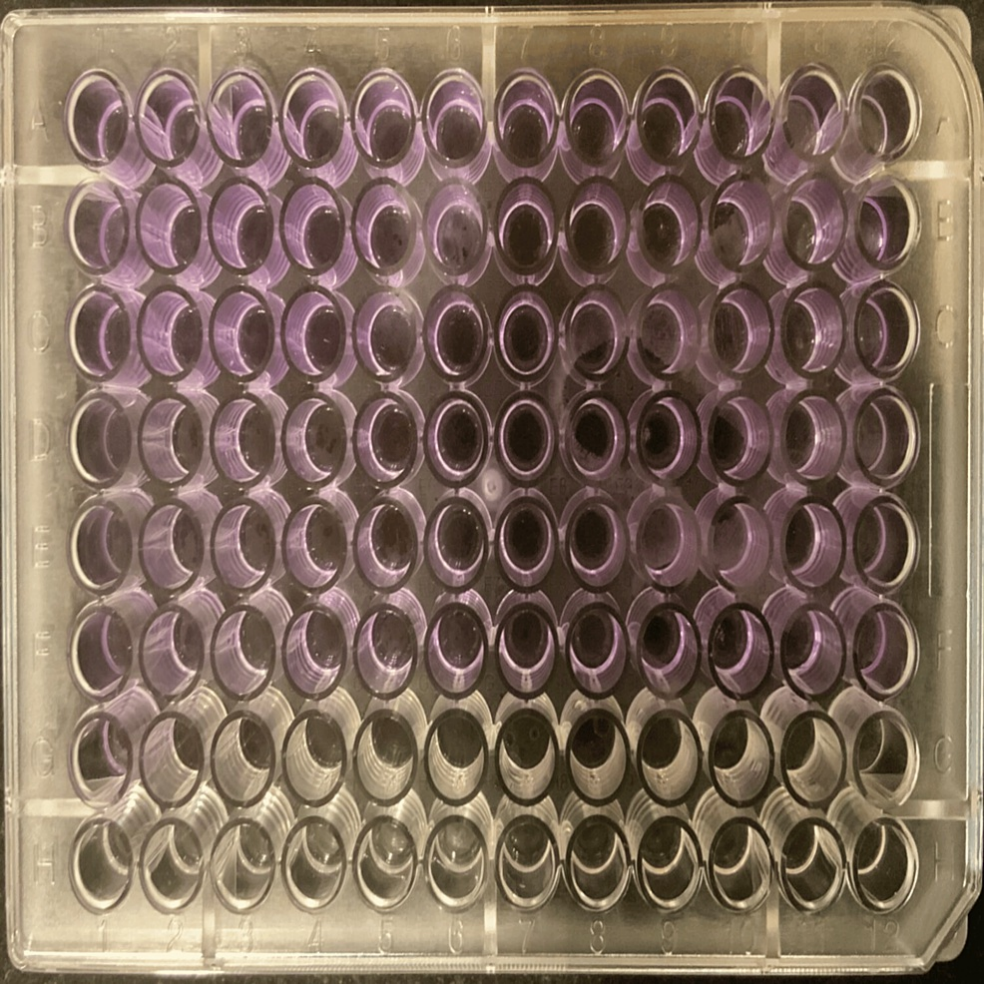
Biofilm formation assessed with crystal violet staining

The crystal violet staining method was used to assess the anti-biofilm properties of leaf extracts of *Terminalia catappa* and *Murraya koenigii* against biofilms produced by *S. mutans *(Figure 1). About 180 μL of the BHI broth medium was mixed with a dose-dependent amount of 20 μL of overnight growth of *S. mutans* and medicinal plants (*Terminalia catappa and Murraya koenigii*) (2, 1, and 0.5 mg/mL), and the mixture was then incubated at 37°C for 24 hours. After washing with sterile water to remove the planktonic cells, the biofilm that had adhered to the surface was stained with a 0.1% crystal violet solution. The unbound crystal violet was rinsed with sterile-distilled water after 15 minutes. An ultraviolet-vis spectrophotometer was used to measure the crystal violet's intensity at 492 nm and 630 nm in order to quantify the adhering biofilm-bound crystal violet that had been eluted in 200 μL of ethanol (95%) [17-22].

## Results

Methanolic crude extract concentrations below the MIC were employed to test for anti-biofilm activity against* S. mutans*. The broth microdilution method was used to assess the MICs of *Murraya koenigii *leaf extracts against* S. mutans*. The MIC of the methanol extract for *S. mutans* was 0.62 mg/ml and below this bacterial growth was observed (Table 1).

**Table 1:** MIC assay for Murraya koenigii against S. mutans MIC: Minimum inhibitory concentration *S. mutans*: *Streptococcus mutans*

S.no.	Methanol extracts	Organism	Conc/ml	MIC
1	Murraya koenigii	Streptococcus mutans	10 mg	MIC
2	Murraya koenigii	Streptococcus mutans	5 mg	MIC
3	Murraya koenigii	Streptococcus mutans	2.5 mg	MIC
4	Murraya koenigii	Streptococcus mutans	1.25 mg	MIC
5	Murraya koenigii	Streptococcus mutans	0.62 mg	MIC
6	Murraya koenigii	Streptococcus mutans	0.31mg	GROWTH
7	Murraya koenigii	Streptococcus mutans	0.15 mg	GROWTH
8	Murraya koenigii	Streptococcus mutans	0.07 mg	GROWTH
9	Murraya koenigii	Streptococcus mutans	0.03 mg	GROWTH
10	Murraya koenigii	Streptococcus mutans	0.015 mg	GROWTH

Same method was used to assess the MIC of the methanolic extracts of *Terminalia catappa* extracts against* S. mutans*. Growth of *S. mutans* was inhibited until 1.25 mg/ml concentrations of the extract and below this concentration growth was observed (Table 2).

**Table 2:** MIC assay for Terminalia catappa against S. mutans MIC: Minimum inhibitory concentration *S. mutans*: *Streptococcus mutans*

S.no.	Methanol extracts	Organism	Conc/ml	MIC
1	Terminalia catappa	Streptococcus mutans	10 mg	MIC
2	Terminalia catappa	Streptococcus mutans	5 mg	MIC
3	Terminalia catappa	Streptococcus mutans	2.5 mg	MIC
4	Terminalia catappa	Streptococcus mutans	1.25 mg	MIC
5	Terminalia catappa	Streptococcus mutans	0.62 mg	GROWTH
6	Terminalia catappa	Streptococcus mutans	0.31 mg	GROWTH
7	Terminalia catappa	Streptococcus mutans	0.15 mg	GROWTH
8	Terminalia catappa	Streptococcus mutans	0.07 mg	GROWTH
9	Terminalia catappa	Streptococcus mutans	0.03 mg	GROWTH
10	Terminalia catappa	Streptococcus mutans	0.015 mg	GROWTH

Effect of medicinal plants (*Murraya koenigii and Terminalia catappa*) on biofilm formation in *S. mutans*

**Figure 2:**
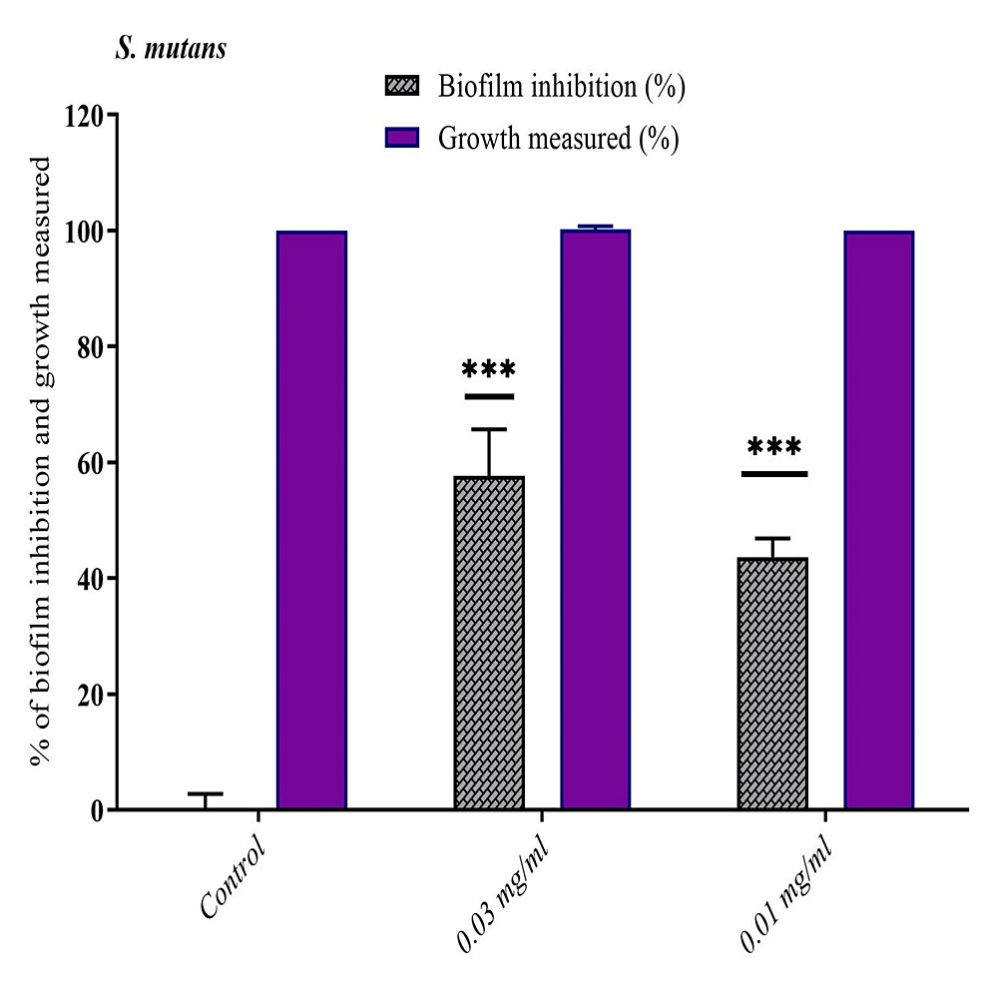
Inhibition of biofilm formation by Murraya koenigii against S. mutans in different concentrations *S. mutans*: *Streptococcus mutans*

The *Murraya koenigii *leaf extracts showed significant reduction in biofilm formation when the organisms were grown in the presence of *S. mutans*. At the lowest concentration of 0.03 mg and 0.01 mg methanolic extract of *Murraya koenigii *has remarkably reduced biofilm formation by 57.6% and 43.6% of *S. mutans,* respectively (Figure 2).

The *Terminalia catappa* leaf extracts did not show any anti-biofilm activity when the organisms were grown in the presence of* S. mutans*.

## Discussion

Medicinal plants have been used for curing and preventing infections since ancient times. Studies have shown that a few phytochemicals from these medicinal plants have anti-quorum sensing and anti-biofilm activity. *Terminalia catappa* leaves contain phytochemicals such as tannins (terflavin A and B, tergallagin, chebulagic acid, graniin, punicalin, punicalagin, and tercatain), saponins, phytosterols, flavonoids. Recently anti-quorum sensing and anti-biofilm properties of *Terminalia catappa* derived flavonoids and tannins have been reported [23-25]. In the present study, an investigation of *Terminalia catappa* and *Murraya koenigii* extract for their in vitro effects of QS-controlled virulence factors and biofilm inhibitory activities against *S. mutans* was performed. The biofilm matrix of carbohydrate polymers works as a food source from which *S. mutans* and other bacteria can extract nutrients by breaking them down. It is interesting to note that in *S. mutans*, a peptide-induced QS system regulates aciduricity and biofilm formation [26,27]. In the present study, *Murraya *koenigii and *Terminalia catappa* leaf extracts inhibited the growth of *S. mutans *at concentrations of 0.62 mg/ml and 1.25 mg/ml, respectively, and *Murraya koenigii *was noted to have a significant anti-biofilm activity against *S. mutans*.

The tannins in* Terminalia catappa* methanolic leaf extracts have been found to have anti-QS and anti-biofilm activity against *Chromobacterium violaceum* and *Pseudomonas aeruginosa* and was evidenced in studies at a concentration of 6.25 mg/ml [23,25]. Reports of anti-biofilm activity of *Terminalia catappa* leaf extract against multi-drug resistant *S. aureus* and *C. albicans* at low concentrations are found [28]. In the present study even though the antibacterial effect of *Terminalia catappa* methanolic extracts was noted against *S. mutans*, no anti-biofilm activity was noted. *Murraya koenigii *has been extensively investigated against common oral pathogens. The essential oil of *Murraya koenigii* showed the most promising QS inhibitory and anti-biofilm activity at a concentration of 0.02% v/v [28]. In the study by Dhamane et al., the antioxidant activity and antibacterial effect of *Murraya koenigii* against *S. mutans* were examined and a significant antibacterial effect at a MIC of 0.625 mg/ml which is very similar to the findings of the present study was noted [8]. In the present study, anti-biofilm effect was evaluated which was not reported previously. Chandra Shekar et al. studied the antibacterial activity of *Murraya koenigii* against *S. mutans* and observed an antibacterial effect comparable to chlorhexidine [10]. In another study by the same author, *Murraya koenigii* was combined with two other medicinal plants, and a very high antibacterial activity was noted against *S. mutans* which was higher than the antibacterial activity of chlorhexidine when used alone [11]. Anti-biofilm activity of certain medicinal plants against* S. mutans* has been studied previously. Belem-Kabré et al. reported that at a concentration of 100 μg/mL, the methanolic extract of *Carica papaya* suppressed 66.10% of the biofilm produced by *S. mutan*s [14]. Murugan et al. reported that the methanol extract of *Achyranthes aspera* (125 g/mL) produced biofilm inhibition of 94% [15]. In this present study,* Murraya koenigii *leaf extracts against *S. mutans* significantly inhibited biofilm formation by 57.6% and 43.6% at the lowest concentrations of 0.03 mg and 0.01 mg, respectively.

Limitations

Limitations of the present study include the use of crude extracts instead of purified compounds. Only one microbe was investigated in this study whereas dental plaque biofilm hosts a variety of microbes. Plant collection was done from one particular location but the constituents may differ when they are grown in different environments and in different seasons. Antioxidant activity also should be evaluated. Further studies may be done involving the late colonizers of dental plaque biofilms. Isolation of the purified compounds from the leaf extracts of these plants can be done and incorporated in oral rinses followed by antimicrobial and antioxidant property evaluation. In vivo studies can be done using the purified compounds of these plant extracts to prevent plaque and biofilm formation. Despite its limitations, the work elucidates a promising antiplaque and antibiofilm chemical that exhibits potential for incorporation into various materials such as mouthwashes, varnishes, and kinds of toothpaste. This can prove to be particularly advantageous in individuals undergoing orthodontic treatment as it can mitigate the occurrence of WSLs and facilitate the maintenance of oral hygiene.

## Conclusions

Considering the limitations of the present in vitro study we can conclude that the leaf extracts of both the studied plant extracts had a good antimicrobial effect against *S. mutans*. The extracts of *Murraya koenigii* had a profound anti-biofilm and QS activity against *S. mutan*s but the leaf extract of *Terminalia catappa* did not have any anti-biofilm activity against* S. mutans*. Hence, *Murraya Koenigii* can be considered for use as a constituent in prophylactic agents.
